# Prevalence and determinants of incident and persistent depressive symptoms among middle-aged and older adults in Thailand: prospective cohort study

**DOI:** 10.1192/bjo.2023.72

**Published:** 2023-05-25

**Authors:** Supa Pengpid, Karl Peltzer, Dararatt Anantanasuwong

**Affiliations:** Department of Health Education and Behavioral Sciences, Faculty of Public Health, Mahidol University, Bangkok, Thailand; Department of Public Health, Sefako Makgatho Health Sciences University, Pretoria, South Africa; and Department of Healthcare Administration, College of Medical and Health Science, Asia University, Taichung, Taiwan; Department of Health Education and Behavioral Sciences, Faculty of Public Health, Mahidol University, Bangkok, Thailand; Department of Psychology, University of the Free State, Bloemfontein, South Africa; and Department of Psychology, College of Medical and Health Science, Asia University, Taichung, Taiwan; Center for Aging Society Research (CASR) at National Institute of Development Administration (NIDA), Bangkapi, Bangkok, Thailand

**Keywords:** Lifestyle factors, chronic diseases, probable depression, prospective cohort study, Thailand

## Abstract

**Background:**

There are no longitudinal studies investigating determinants of incident and persistent depressive symptoms in Southeast Asia.

**Aims:**

To estimate the proportion and correlates of incident and persistent depressive symptoms in a prospective cohort study among middle-aged and older adults (≥45 years) in Thailand.

**Method:**

We analysed longitudinal data from the Health, Aging, and Retirement in Thailand (HART) surveys in 2015 and 2017. Depressive symptoms were assessed using the Center for Epidemiologic Studies Depression Scale. Logistic regression was used to calculate predictors of incident and persistent depressive symptoms.

**Results:**

In total, 290 of 4528 participants without depressive symptoms in 2015 had incident depressive symptoms in 2017 (9.8%) and 76 of 640 adults had persistent depressive symptoms (in both 2015 and 2017) (18.3%). In adjusted logistic regression analysis, having diabetes (adjusted odds ratio AOR = 1.48, 95% CI 1.07–2.05), musculoskeletal conditions (AOR = 1.56, 95% CI 1.01–2.41) and having three or more chronic conditions (AOR = 2.55, 95% CI 1.67–3.90) were positively associated and higher subjective economic status (AOR = 0.47, 95% CI 0.31–0.72) and social participation (AOR = 0.66, 95% CI 0.49–0.90) were inversely associated with incident depressive symptoms. Having a cardiovascular disease (AOR = 1.55, 95% CI 1.01–2.39) and having three or more chronic conditions (AOR = 2.47, 95% CI 1.07–5.67) were positively associated and social participation (AOR = 0.48, 95% CI 0.26–0.87) was inversely associated with persistent depressive symptoms.

**Conclusions:**

One in ten middle-aged and older adults had incident depressive symptoms at 2-year follow-up. The prevalence of incident and/or persistent depression was higher in people with a lower subjective economic status, low social participation, diabetes, musculoskeletal disorders, cardiovascular conditions and a higher number of chronic diseases.

Depressive disorders are the most prevalent mental health problems in the general population.^1^ In community studies from 30 countries the point prevalence of depression has been estimated at 12.9%.^2^ Among middle-aged and older adults in six low- and middle-income countries, the prevalence of depression was 7.5%, with the highest rate in India (15.2%),^3^ and among ageing adults 27.9% had depressive symptoms in China^4^ and 11.5% in Malaysia.^5^ In Thailand, in the general adult population, 2.5% had a major depressive disorder;^6^ and among older adults 18.5% had depressive symptoms in Chachoengsao Province^7^ and 28.5% in Kanchanaburi.^8^ Late-life depression is associated with various negative consequences, including impairment in social functioning, reduced quality of life, increased comorbidity, lower medication adherence and increased suicidal behaviour.^9^

Owing to a demographic and epidemiological transition in Thailand, non-communicable diseases, including mental disorders such as depression, have become more prevalent.^10–12^ Considering that previous studies on depression in Thailand were cross-sectional, the prevalence of incident and persistent depressive symptoms among middle-aged and older adults in Thailand is unclear, as are the prospective relationships between baseline indicators and incident and persistent depressive symptoms. A greater understanding of the prevalence of incident and persistent depressive symptoms and of the factors associated with their occurrence may help in better identifying and addressing modifiable risk factors in the population.

Various longitudinal studies have identified health indicators associated with incident and/or persistent depression in middle-aged and older adults, including lifestyle factors (smoking, heavy alcohol use),^13^ physical inactivity,^13,14^ body weight^15,16^ and specific chronic diseases, such as stomach/digestive diseases,^9,17^ diabetes,^9,18^ arthritis/rheumatism,^9,18–20^ liver disease,^19^ kidney disease,^9,17,19,21^ sensory loss,^18^ hypertension,^18,22^ cardiovascular disease,^18,19,23,24^ chronic lung disease,^9,17,18^ mild cognitive impairment and dementia,^25^ memory-related disease^26^ and cancer.^19^ A higher number of chronic diseases was associated with a higher risk of incident depression.^17,27^ Other risk factors for depression may include low social support, adverse life events, and biological and sociodemographic factors.^28–30^ There is a lack of longitudinal studies in Southeast Asia investigating determinants of incident and persistent depressive symptoms. To address this research gap, our objective was to investigate the prevalence of incident and persistent depressive symptoms and factors associated with their occurrence in a prospective cohort study among ageing adults (≥45 years) in Thailand.

## Method

### Sample and procedure

We analysed longitudinal data from two waves (2015 and 2017) of the Health, Aging and Retirement in Thailand (HART) study. In a three-stage (region, province, blocks or villages) stratified random sampling in each household, one person (≥45 years) was randomly selected. For frail respondents proxy interviews were administered.^31,32^ In the 2015 (*n* = 5616) and the 2017 surveys (*n* = 3708) the response and retention rates were 72.3% and 66.0% respectively; at follow-up 192 had died, 1554 had moved away from the study area and 270 declined participation.

Participants were interviewed using a structured questionnaire in 2015 and using computer-assisted personal interviewing (CAPI) in 2017. The study was approved by the Ethics Committee in Human Research at the National Institute of Development Administration – ECNIDA (ECNIDA 2020/00012) and participants gave their written informed consent.

### Measures

#### Outcome variable

Participants completed the Center for Epidemiologic Studies Depression Scale (CES-D-10), and scores ≥10 were defined as indicating the presence of depressive symptoms.^33^ The CES-D-10 is valid in Thai adult populations.^34,35^ The internal consistency of the CES-D-10 in the study population ranged from 0.72 in 2017 to 0.78 in 2015.

#### Covariates

Sociodemographic variables included education, marital status, gender, age, education, religion and subjective economic status.

Substance use included alcohol use and smoking (tobacco use), rated as never, past or current.

Physical activity was classified as 0–149 min/week exercise and ≥150 min/week exercise.^36,37^

Body mass index (BMI), calculated from self-reported height and weight, was stratified as: underweight (<18.5 kg/m^2^), normal weight (18.5–22.9 kg/m^2^), overweight (23–24.9 kg/m^2^) and obesity (≥25 kg/m^2^).^38^

Social participation (at least one social activity in the past month) was sourced from six items.^39^

Participants were asked about 12 conditions diagnosed by a healthcare provider: hypertension; diabetes; vascular diseases, heart disease or heart failure; rheumatism or arthritis; bone diseases, low bone density or osteoporosis; kidney disease; lung disease/emphysema; cancer; liver disease; Alzheimer's disease/brain diseases; visual impairment; and hearing impairment. The 12 chronic diseases were classified into 8 groups: (a) cardiovascular: hypertension, heart disease, cardiovascular disease, heart failure; (b) endocrine (diabetes); (c) musculoskeletal (arthritis/rheumatism, osteoporosis and bone diseases); (d) liver or kidney disease; (e) respiratory (lung disease/emphysema); (f) cancer; (g) sensory (visual impairment and/or hearing impairment); and (h) neurological (brain diseases/Alzheimer's disease).

### Statistical analysis

Frequencies and percentages of incident and persistent depressive symptoms were calculated. The first longitudinal logistic regression model estimated incident depressive symptoms in 2017, excluding those with depressive symptoms in 2015, and the second model estimated persistent depressive symptoms (in both 2015 and 2017). Models were adjusted by chronic diseases, sociodemographic factors, lifestyle factors, social participation and BMI; confounders were included based on literature review.^9,17^ *P* ≤ 0.05 was considered statistically significant. Missing data were discarded. Statistical analyses were conducted with Stata SE version 15.0 for Windows.

## Results

### Sample characteristics

In total, 290 of 4528 participants without depressive symptoms in 2015 had incident depressive symptoms in 2017 (9.8%), and 76 of 640 adults had persistent depressive symptoms (in both 2015 and 2017) (18.3%). The details of the sample are shown in Table 1.

**Table 1:** Sample characteristics by incident and persistent depressive symptoms, Thailand, 2015–2017

Baseline variables	Subcategories	Baseline sample	Incident depressive symptoms	Persistent depressive symptoms
		*n* (%)	*n* (%)	*n* (%)
All		5616	290 (9.8)	76 (18.3)
Age, in years	45–54	1105 (19.7)	51 (9.0)	9 (15.0)
	55–64	1500 (26.7)	61 (7.5)	17 (16.2)
	66–74	1370 (24.4)	83 (10.8)	21 (20.0)
	75 or more	1641 (29.2)	95 (11.4)	29 (19.9)
Gender	Female	2930 (52.2)	164 (10.5)	49 (19.7)
	Male	2686 (47.8)	126 (8.9)	27 (16.2)
Education	None	363 (6.5)	16 (10.1)	8 (13.6)
	Elementary	4217 (75.4)	237 (10.3)	62 (20.0)
	>Elementary	1016 (18.2)	35 (7.0)	6 (13.0)
Marital status	Not married	2352 (41.9)	143 (11.8)	29 (15.8)
	Married/cohabiting	3258 (58.1)	147 (8.3)	47 (20.3)
Religion	Muslim or other	401 (7.1)	25 (12.3)	9 (11.5)
	Buddhist	5208 (92.9)	565 (9.6)	67 (19.9)
Subjective economic status	Low	503 (9.3)	41 (15.9)	20 (28.6)
	Middle	3136 (57.9)	173 (10.4)	39 (17.1)
	High	1777 (32.8)	72 (7.3)	13 (13.5)
Social participation	No	3957 (70.6)	228 (10.8)	58 (21.8)
	Yes	1650 (29.4)	62 (7.1)	18 (12.1)
Alcohol use	Never	4530 (80.7)	237 (9.9)	67 (19.3)
	Past	391 (7.0)	23 (11.0)	6 (18.2)
	Current	695 (12.4)	30 (8.1)	3 (8.6)
Smoking (tobacco use)	Never	4483 (79.8)	234 (9.8)	63 (18.8)
	Past	426 (7.6)	23 (10.2)	7 (20.0)
	Current	707 (12.6)	33 (9.0)	6 (13.3)
Physical activity	≥150 min/week	877 (15.6)	48 (8.7)	12 (24.5)
	<150 min/week	4739 (84.4)	245 (10.0)	64 (17.4)
Body mass index	Normal	1912 (37.7)	113 (11.2)	22 (15.2)
	Underweight	559 (11.0)	26 (9.6)	8 (14.5)
	Overweight	1007 (19.9)	46 (8.2)	8 (14.8)
	Obesity	1592 (31.4)	82 (9.5)	24 (21.6)
Chronic conditions				
Cardiovascular	No	3554 (63.3)	169 (8.9)	35 (16.5)
	Yes	2062 (36.7)	121 (11.3)	41 (20.1)
Endocrine (diabetes)	No	4767 (84.9)	230 (9.1)	62 (18.5)
	Yes	849 (15.1)	60 (13.8)	14 (17.5)
Musculoskeletal	No	5249 (93.5)	262 (9.4)	65 (17.4)
	Yes	367 (6.5)	28 (14.6)	11 (25.6)
Liver or kidney disease	No	5491 (97.8)	281 (9.7)	74 (18.4)
	Yes	125 (2.2)	9 (13.6)	2 (14.3)
Respiratory	No	5567 (99.1)	286 (9.7)	73 (17.8)
	Yes	49 (0.9)	4 (14.8)	3 (42.9)
Cancer	No	5565 (99.1)	285 (9.7)	75 (18.2)
	Yes	51 (0.9)	5 (19.2)	1 (25.0)
Sensory	No	4842 (86.2)	239 (9.3)	53 (16.4)
	Yes	774 (13.8)	51 (12.7)	23 (25.0)
Neurological	No	5569 (99.2)	286 (9.7)	74 (18.1)
	Yes	47 (0.8)	4 (25.0)	2 (25.0)
Number of chronic conditions				
Chronic conditions	0	2720 (48.4)	111 (7.6)	18 (13.1)
	1	1617 (28.8)	89 (10.3)	26 (19.4)
	2	898 (16.0)	56 (12.0)	16 (18.0)
	3 or more	381 (6.8)	34 (18.1)	16 (28.6)

### Associations with incident depressive symptoms

In adjusted logistic regression analysis, having diabetes (adjusted odds ratio AOR = 1.48, 95% CI 1.07–2.05), musculoskeletal conditions (AOR = 1.56, 95% CI 1.01–2.41) and having three or chronic conditions (AOR = 2.55, 95% CI 1.67–3.90) were positively associated and a higher subjective economic status (AOR = 0.47, 95% CI 0.31–0.72) and social participation (AOR = 0.66, 95% CI 0.49–0.90) were inversely associated with incident depressive symptoms. In addition, in the unadjusted analysis, cardiovascular, sensory and neurological conditions were positively associated with incident depressive symptoms (Table 2).

**Table 2:** Odds ratios for the association between chronic conditions and incident depressive symptoms, Thailand, 2015–2017

Baseline variables	Subcategory	COR (95% CI)	AOR (95% CI)^a^
Age, years	45–54	1 (Reference)	–
	55–64	0.83 (0.56–1.22)	
	66–74	1.23 (0.85–1.78)	
	75 or more	1.31 (0.92–1.88)	
Gender	Female	1 (Reference)	–
	Male	0.84 (0.66–1.07)	
Education	None	1 (Reference)	–
	Elementary	1.02 (0.60–1.74)	
	>Elementary	0.67 (0.36–1.25)	
Marital status	Not married	1 (Reference)	–
	Married/cohabiting	1.37 (0.82–2.27)	
Religion	Muslim or other	1 (Reference)	–
	Buddhist	1.90 (0.90–4.01)	
Subjective economic status	Low	1 (Reference)	1 (Reference)
	Middle	0.62 (0.43–0.89)**	0.67 (0.46–0.98)*
	High	0.42 (0.28–0.63)***	0.47 (0.31–0.72)***
Social participation	No	1 (Reference)	1 (Reference)
	Yes	0.63 (0.47–0.85)**	0.66 (0.49–0.90)**
Alcohol use	Never	1 (Reference)	–
	Past	1.12 (0.71–1.76)	
	Current	0.80 (0.54–1.19)	
Smoking (tobacco use)	Never	1 (Reference)	–
	Past	1.04 (0.66–1.63)	
	Current	0.91 (0.62–1.33)	
Physical activity	≥150 min/week	1 (Reference)	
	<150 min/week	1.16 (0.83–1.61)	–
Body mass index	Normal	1 (Reference)	–
	Underweight	0.84 (0.54–1.32)	
	Overweight	0.71 (0.50–1.02)	
	Obesity	0.83 (0.61–1.12)	
Chronic conditions			
Cardiovascular	No	1 (Reference)	1 (Reference)
	Yes	1.34 (1.09–1.65)**	1.15 (0.92–1.45)
Endocrine (diabetes)	No	1 (Reference)	1 (Reference)
	Yes	1.60 (1.18–2.17)**	1.48 (1.07–2.05)*
Musculoskeletal	No	1 (Reference)	1 (Reference)
	Yes	1.68 (1.10–2.56)*	1.56 (1.01–2.41)*
Liver or kidney disease	No	1 (Reference)	–
	Yes	1.48 (0.73–3.03)	
Respiratory	No	1 (Reference)	
	Yes	1.63 (0.56–4.73)	
Cancer	No	1 (Reference)	
	Yes	2.23 (0.84–5.59)	
Sensory	No	1 (Reference)	1 (Reference)
	Yes	1.44 (1.04–1.99)*	1.24 (0.88–1.73)
Neurological	No	1 (Reference)	1 (Reference)
	Yes	3.41 (1.08–10.69)*	2.47 (0.74–8.25)
Number of chronic conditions			
Chronic conditions	0	1 (Reference)	1 (Reference)^a^
	1	1.38 (1.03–1.84)*	1.37 (1.02–1.84)*
	2	1.67 (1.19–2.35)**	1.60 (1.13–2.16)**
	3 or more	2.70 (1.78–4.11)***	2.55 (1.67–3.90)***

COR, crude odds ratio; AOR, adjusted odds ratio.

a. adjusted for all variables except for individual chronic conditions.

* *P* < 0.05, **P < 0.01, ***P < 0.001.

### Associations with persistent depressive symptoms

In adjusted logistic regression analysis, having a cardiovascular condition (AOR = 1.55, 95% CI 1.01–2.39) and having three or more chronic conditions (AOR = 2.47, 95% CI 1.07–5.67) were positively associated and social participation (AOR = 0.48, 95% CI 0.26–0.87) was negatively associated with persistent depressive symptoms. In addition, in univariable analysis, higher subjective economic status was negatively associated with persistent depressive symptoms (Table 3).

**Table 3:** Odds ratios for the association between chronic conditions and persistent depressive symptoms, Thailand, 2015–2017

Baseline variables	Subcategory	COR (95% CI)	AOR (95% CI)
Age, years	45–54	1 (Reference)	–
	55–64	1.10 (0.46–2.64)	
	66–74	1.42 (0.60–3.33)	
	75 or more	1.41 (0.62–3.18)	
Gender	Female	1 (Reference)	–
	Male	0.79 (0.47–1.32)	
Education	None	1 (Reference)	–
	Elementary	1.59 (0.72–3.53)	
	>Elementary	0.96 (0.31–2.98)	
Marital status	Not married	1 (Reference)	–
	Married/cohabiting	1.37 (0.82–2.27)	
Religion	Muslim or other	1 (Reference)	–
	Buddhist	1.90 (0.90–3.01)	
Subjective economic status	Low	1 (Reference)	1 (Reference)
	Middle	0.52 (0.28–0.96)*	0.59 (0.30–1.14)
	High	0.39 (0.18–0.86)*	0.46 (0.21–1.05)
Social participation	No	1 (Reference)	1 (Reference)
	Yes	0.49 (0.28–0.87)*	0.48 (0.26–0.87)*
Alcohol use	Never	1 (Reference)	–
	Past	0.93 (0.37–2.35)	
	Current	0.39 (0.12–1.32)	
Smoking (tobacco use)	Never	1 (Reference)	–
	Past	1.08 (0.45–2.59)	
	Current	0.67 (0.27–1.64)	
Physical activity	≥150 min/week	1 (Reference)	
	<150 min/week	0.65 (0.32–1.32)	–
Body mass index	Normal	1 (Reference)	–
	Underweight	0.95 (0.40–2.29)	
	Overweight	0.97 (0.40–2.34)	
	Obesity	1.54 (0.81–2.93)	
Chronic conditions			
Cardiovascular	No	1 (Reference)	1 (Reference)
	Yes	1.64 (1.08–2.49)*	1.55 (1.01–2.39)*
Endocrine (diabetes)	No	1 (Reference)	–
	Yes	1.01 (0.53–1.93)	
Musculoskeletal	No	1 (Reference)	
	Yes	1.85 (0.88–3.89)	
Liver or kidney disease	No	1 (Reference)	
	Yes	0.42 (0.05–3.30)	
Respiratory	No	1 (Reference)	–
	Yes	2.39 (0.43–13.28)	
Cancer	No	1 (Reference)	–
	Yes	1.57 (0.16–15.34)	
Sensory	No	1 (Reference)	–
	Yes	1.66 (0.94–2.96)	
Neurological	No	1 (Reference)	–
	Yes	1.90 (0.36–10.01)	
Number of chronic conditions			
Chronic conditions	0	1 (Reference)	1 (Reference)
	1	1.72 (0.87–3.40)	1.74 (0.86–3.52)
	2	1.63 (0.77–3.46)	1.49 (0.68–3.29)
	3 or more	2.94 (1.33–6.40)**	2.47 (1.07–5.67)*

COR, crude odds ratio; AOR, adjusted odds ratio.

* *P* < 0.05, **P < 0.01.

## Discussion

In this first prospective cohort study among middle-aged and older adults in Thailand, we found that the prevalence of incident depressive symptoms at 2-year follow-up was 9.8%, which is lower than the prevalence among middle-aged and older adults in China reported in a 4-year follow-up study (22.3%)^9^ and lower than cross-sectional rates of depressive symptoms (18.5–28.5%) among older adults reported in local studies in Thailand.^7,8^ This study showed that depressive symptoms are a significant public health issue in Thailand, calling for intervention programmes to reduce the burden of depressive symptoms.

We found that lower subjective economic status, low social participation, diabetes, musculoskeletal conditions and a higher number of chronic conditions were associated with incident depressive symptoms. Low social participation, cardiovascular conditions and a higher number of chronic conditions were associated with persistent depressive symptoms. The observed associations were similar across genders, age, education, marital status and religion.

Previous research^9,18^ has shown, as in this study, that diabetes is associated with incident depression. This can be explained by the fact that there is currently no cure for diabetes and that individuals are required to control the condition by adhering to medication and strict diets, which in turn may lead to increased negative emotions.^17^ Consistent with previous studies,^9,18–20^ this study found an association between musculoskeletal conditions and incident depressive symptoms. Several factors may be responsible for this association, including the absence of a cure for the musculoskeletal condition, the interference of pain with daily activities, medication side-effects and shared risk factors for inflammation for both conditions.^9^

Furthermore, in line with previous studies,^18,19,22–24^ we found a positive association between cardiovascular disease and persistent depressive symptoms. Previous research showed a bidirectional association between persistent depression and cardiovascular disease,^40^ which may explain our findings. In univariable analysis, we also found an association between cardiovascular disease, sensory impairment and neurological (brain diseases/Alzheimer's disease) conditions and incident depressive symptoms, which is consistent with previous research.^9,18,22–26^ Ageing adults with impaired vision and/or hearing may be more likely to experience functional disability and poor social support, which can lead to incident depression.^18^ In our study, ageing adults with brain diseases/Alzheimer's disease had a high prevalence of incident and persistent depressive symptoms (25.0%), which is similar to a large study among older adults in the USA, which found that at 2-year follow-up 25% of participants with dementia and 22% of those with mild cognitive impairment had developed depression.^25^ It is suggested that depression develops as a comorbid condition during the course of dementia, necessitating integrated management of both dementia and depression.^25^ Contrary to what was found previously,^9,17–19,21^ we did not find an association between liver disease, kidney disease, lung disease, cancer and incident and persistent depressive symptoms.

In accordance with previous research,^13,17,27^ we found an association between an increasing higher number of chronic diseases and incident and persistent depressive symptoms. Having several comorbid chronic diseases may have a negative effect on various body organs, increase symptom burden and disability, and require lifelong treatment, all of which may contribute to an increase in negative emotions, leading to incident depressive symptoms.^17,19^ This finding highlights the relevance of attending to mental health effects in diagnoses and management of multiple chronic conditions.^19^

Unlike some previous research,^13–16^ we did not find a significant association between smoking, alcohol use, physical inactivity or body weight and incident and persistent depressive symptoms. Furthermore, we did not find significant gender and age differences in the prevalence of depressive symptoms, whereas some other studies^9^ found a preponderance of incident depressive symptoms among women and a decline with age.

### Study limitations

A study limitation was the high loss to follow-up (32%). This reduced the sample of those with persistent depressive symptoms, resulting in larger confidence intervals. We lack information on survival bias and other information on participants lost to follow-up, which reduces the generalisability of the results. Furthermore, the study used a screening questionnaire for depression. Future research should at least on a subsample perform a diagnostic psychiatric evaluation. Diagnosis of depression is especially relevant in the context of comorbidity with diabetes and multi-morbidity, as there is a risk of significant diagnostic overshadowing. For example, a person with poor diabetes control may have changes in appetite, sleep and energy levels associated with hyperglycaemia, which is a further limitation of the study. The follow-up period (2 years) was relatively short and longer repeated follow-ups may be needed to identify stronger results.

### Implications

Our results show the importance of baseline health status indicators in predicting longitudinal changes in depressive symptoms. Identifying individuals with the identified risk factors can help in providing early interventions to prevent the development of depression.

## Data Availability

The study data are publicly available from the Gateway to Global Aging Data platform: Health, Aging, and Retirement in Thailand (HART) study at https://g2aging.org/?section = study&studyid = 44. (Please note that the year for Wave 2 on the Gateway to Global Aging Data website mistakenly states '2016'; we confirm this is actually the 2017 data used in this paper.)
